# Acoustic vector sensor analysis of the Monterey Bay region soundscape and the impact of COVID-19a)

**DOI:** 10.1121/10.0010162

**Published:** 2022-04-12

**Authors:** Kevin B. Smith, Paul Leary, Thomas Deal, John Joseph, John Ryan, Chris Miller, Craig Dawe, Benjamin Cray

**Affiliations:** 1 Naval Postgraduate School, Monterey, California 93943, USA; 2 Naval Undersea Warfare Center, Newport, Rhode Island 02841, USA; 3 Monterey Bay Aquarium Research Institute, Moss Landing, California 95039, USA

## Abstract

From February 2019 through January 2021, data were collected by an acoustic vector sensor moored on the seafloor at a depth of approximately 900 m just outside of Monterey Bay, California, near a major shipping lane off the California coast. Analysis of the vector sensor data has shown the ability to accurately determine bearings to merchant vessels at ranges up to 60 km. This paper examines the features of the low-frequency soundscape using spectral probability densities and evaluates directional features through vector intensity processing as well as coherent linear and adaptive processing of the vector sensor channels. Merchant vessel acoustic data were analyzed using the 1/3 octave band centered at 63 Hz. Over the period analyzed, a reduction in merchant vessel noise was observed between February and June 2020 relative to the same period in 2019, consistent with a reduction in vessel traffic due to the worldwide response to COVID-19. The directional features of the data evaluated through adaptive processing methods also suggest this reduction can be most clearly distinguished towards the south, where the shipping lane is limited to transiting vessels, rather to the north-northwest, where merchant vessels tend to congregate on approach into the San Francisco Bay area.

## I. INTRODUCTION

Numerous studies have reported a reduction in merchant shipping traffic and other marine vessel activity around the world in response to the COVID-19 pandemic.^1–3^ This has been mostly highlighted in transcontinental shipping carrying goods and products between Asia and North America. Major ports along the west coast of the United States, such as Long Beach, CA, Oakland, CA, and Seattle, WA have been directly affected by the reduced merchant traffic.

Such reductions should be accompanied by a coincidental reduction in related ambient noise levels recorded on underwater acoustic sensors. A recent paper by Thomson and Barclay^4^ examined long-term acoustic data recordings from the NEPTUNE and VENUS cabled observatories near Vancouver, Canada. They combined analysis of trade activity, Automatic Identification System (AIS) shipping data, and acoustic data to understand changes in ambient noise. They focused their analysis on signal levels at 100 Hz. While the results suggested potential reductions in ambient noise due to the impact of COVID-19 on reduced merchant traffic, they also found that overall weekly average noise levels were reduced in 2020 as compared to 2019 by as much as 1.5 dB, implying other factors were contributing. A similar study using a cabled observatory off central California linked reduction of low-frequency noise during the first half of 2020 to reduced shipping activity, as characterized from both AIS vessel tracking data and economic data from all California ports.^5^

In the seminal work by Wenz,^6^ features of ambient noise curves were established (so-called Wenz curves) which indicated that distant shipping was the dominant contributor to ambient noise levels below 100 Hz. Above this frequency, local meteorological conditions (wind and breaking waves) were expected to dominate the ambient noise contributions. The text by Carey and Evans^7^ also refers to several experimental observations which found wind and breaking waves could have significant influence on the ambient soundscape down to frequencies as low as 50 Hz.

In this paper, data collected from a single vector sensor deployed off the central coast of California, just outside Monterey Bay, were examined in an effort to relate ambient noise level variations in merchant shipping activity. This region falls within the Monterey Bay National Marine Sanctuary, and there are no major industrial ports present.^8^ The majority of marine activity is limited to local fishing fleets, whale-watching vessels, various research vessels, and pleasure craft. The primary harbors utilized in Monterey Bay include those at Moss Landing and Monterey with a smaller presence in Santa Cruz. On the western edge of the Sanctuary boundary is a primary west coast shipping lane which supports merchant vessel traffic between Long Beach/Southern California and San Francisco/Oakland.^9^

The Monterey Accelerated Research System (MARS) is a cabled observatory situated approximately 22 miles due west of Moss Landing California at 890 m water depth. It was first deployed in 2008 by the Monterey Bay Aquarium Research Institute (MBARI) as a test bed for larger scale efforts now deployed around the country and around the world.^10^ In recent years, a large focus has been on acoustic studies with hydrophones listening to biological and human generated sound, and echo sounders probing the water for organisms and oceanographic features.

In order to focus our analysis on the impact of merchant shipping variations, data were examined initially across a broad frequency spectrum, 25–400 Hz, and long-time scales over two years. Directional processing of the vector sensor data were also employed to distinguish features of interest. Statistical probabilities of power spectral densities were then used as measures to evaluate the impact of the COVID-19 reductions in merchant vessel noise levels.

Section II of this paper presents a thorough justification of the data analysis methods used to produce the final results. The impacts of wind noise on both spectral levels and directional estimates across the band are highlighted. The use of statistical probabilities of power spectral densities is introduced as a fundamental measure of analysis, and annual variations of these statistics are reviewed to help establish the justification for the final processing approach. A theoretical hypothesis and practical application on the limitations of directional processing from a single vector sensor is presented, leading to recommendations on the use of such data in long-term statistics. Section III presents the final results utilizing the specific processing approaches outlined. The impact of COVID-19 shipping traffic reductions on the directional ambient soundscape is then clearly articulated.

## II. DATA ANALYSIS METHODS

**FIG. 1.**
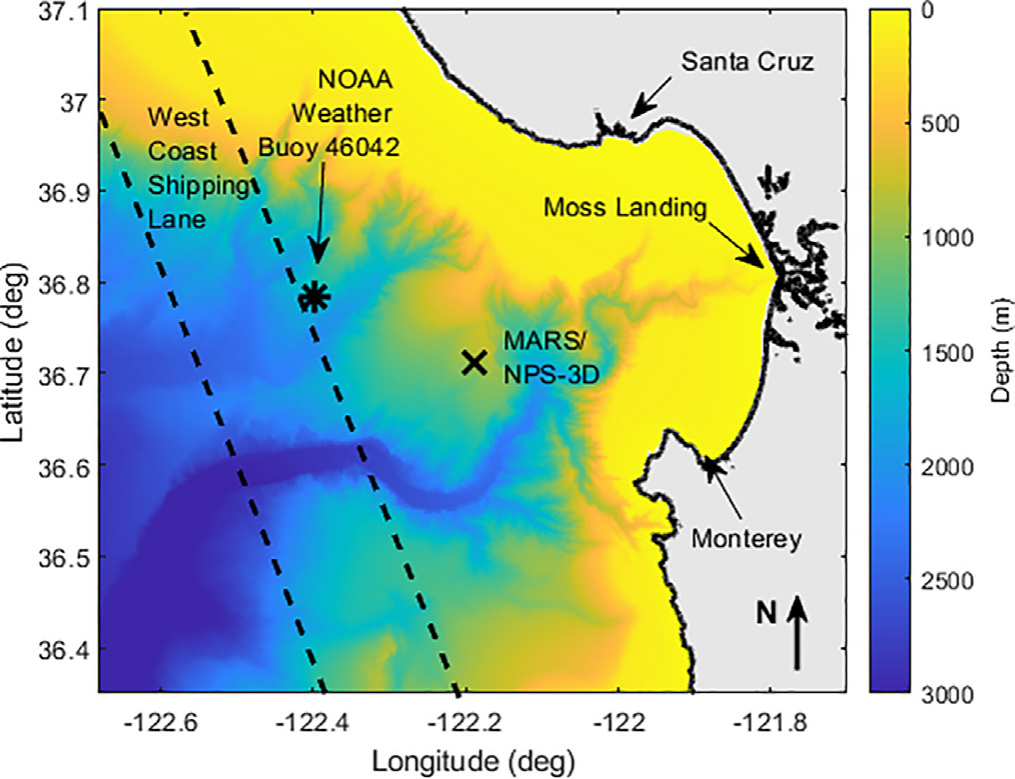
(Color online) Bathymetric map of the Monterey Bay region. Markers indicate the location of the MARS/NPS-3D sensor location (**X**) and NOAA Weather Buoy No. 46042 (*). Dashed lines to the west indicate primary shipping lane.

The majority of the acoustic data processed for this study was collected by a Naval Postgraduate School (NPS) directional acoustic sensor (vector sensor) developed by Geospectrum Technologies, Inc (GTI). This particular GTI M20 sensor system was configured for deployment on the MARS observatory. The sensor system, designated on the MARS observatory as the NPS-3D node, was deployed by MBARI near the MARS site just outside Monterey Bay on January 31, 2019, at a depth of 891 m. Figure 1 depicts the location of the MARS/NPS-3D sensor location, represented by an “X” near the center of the plot. Locations of the primary marine harbors are also indicated, along with the general range of the west coast shipping lane extending approximately 15–30 km from the sensor at the Closest Point of Approach (CPA). The location of a weather buoy, managed by the National Oceanographic and Atmospheric Administration (NOAA), is also indicated in the figure. Data collected on wind speed is evaluated and associated with its impact on ambient noise levels observed on the NPS-3D node.

Data from the sensor are streamed in near-real-time to a server at NPS, and have been archiving almost continuously since its deployment. The one critical data failure since it began operating was during the months of March and April 2020. Still, these data provide unique perspectives on directional acoustic data at the onset of COVID-19 responses and continue to provide current data for comparison with annual trends. As will be seen, the impact on shipping noise has not been limited to early 2020. To fill the recording gap of the NPS-3D sensor, recordings from an omnidirectional hydrophone co-deployed on the MARS observatory were used for March and April 2020. Data collected from other months were used to confirm similar calibration levels from the two systems. The specifics of this sensor are described in Ref. 11.

### A. Data description

The uniqueness of the MARS/NPS-3D data is due to the vector sensor system employed. The directional acoustic data consists of four channels sampled at 8 kHz—one omnidirectional (pressure) channel and three orthogonally oriented dipole (acceleration) channels. Each channel has a specific calibration curve, as well as calibrated phase information of the three dipole channels relative to the omnidirectional channel. The operational band of the sensor, based on the provided calibration data, is typically limited from 20 to 1200 Hz.

Data processing scripts developed at NPS utilize 1 s of data with 50% overlap between samples to produce spectrogram data in 1 Hz bins every 0.5 s, consistent with Welch's method.^12^ A Hann window is used over each 1 s data chunk, providing an effective noise bandwidth (ENBW) of 1.5 dB.^13^ After transformation of each channel to the frequency domain, calibration values are applied to produce complex spectral data for pressure and three components of particle velocity.

**FIG. 2.**
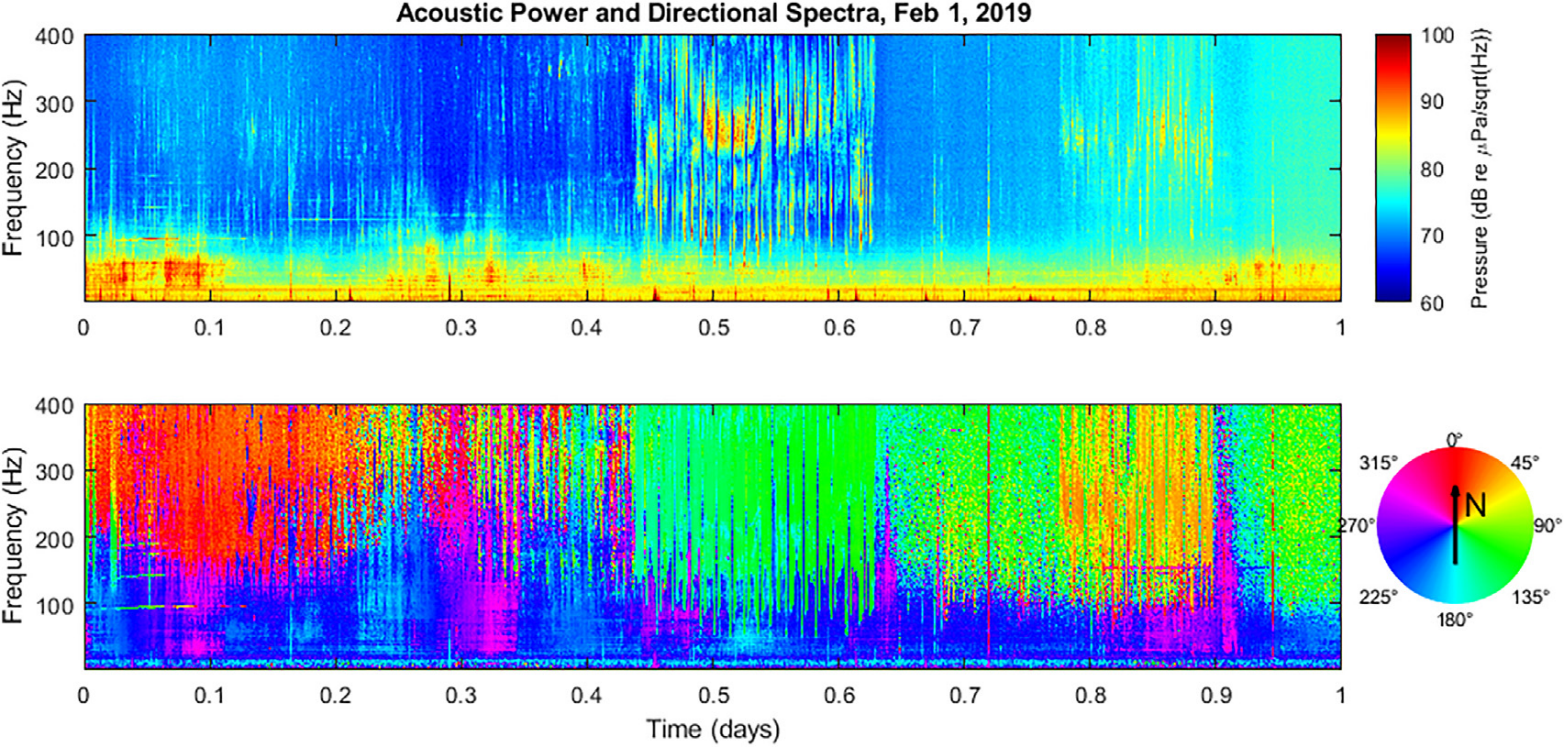
(Color online) Standard acoustic power spectra (upper plot) and directional spectra (lower plot) based on processed data from February 1, 2019. Note directional colorbar rosette used to indicate signal bearings in lower plot.

By multiplying the horizontal velocity components with the pressure data, standard intensity processing^14,15^ of the complex acoustic intensity allows for bearing estimation within each spectrogram bin. An example one-day energy spectrogram and directional spectrogram are displayed in Fig. 2 for the entire day of February 1, 2019, the first full day of operation of the sensor system. The frequencies displayed range from 25 to 400 Hz. The timescale of data records stored on the server are in UTC units, so the beginning of the record, 00:00:00 UTC on February 1, corresponds to a local time of 16:00:00 PST on January 31 (the afternoon after the system was deployed). Time scales presented here have been converted to units of days for simplicity (with February 1 running from 0 to 1 days). Directional spectrograms use an “hsv” color palette wheel to indicate bearings since the color palette forms a continuous transition around 360º.

Near the beginning of the record, there is an extended period of humpback vocalizations in the 100–400 Hz band that appear to be coming from a northerly bearing. In the middle of the record between about 0.45 and 0.62 days, there is another extended period of loud humpback vocalizations to the ESE of the MARS site. And near the end of the record between about 0.77 and 0.90 days, there is another group of humpback vocalizations from the NE. A loud, impulsive signal is also observed around 0.72 days coming from the NW.

This plot also highlights the distant shipping traffic that dominates the acoustic spectrum below about 100 Hz. Signals are observed to pass from the SW to the NW, or vice versa. Some signals are also detected nearly due south. It is down in this lower frequency band that much of the effects from a COVID-19 response would be expected to be observable due to reduced shipping activity between 2019 and 2020.

In order to focus our analyses on signal levels due to shipping, an appropriate frequency band needs to be defined. As previously noted, the Wenz curves typically characterize shipping noise as dominant below 100 Hz. In addition, the Monterey Bay is known to be visited by numerous migratory whales, including humpbacks, blues, and fins. Humpback vocalizations tend to be above 100 Hz, and should not bias results below this limit. Blue whales vocalize largely below 100 Hz, while fin whale vocalizations are largely below 30 Hz. A detailed review of other ambient noise effects is required.

### B. Effect of wind

**FIG. 3.**
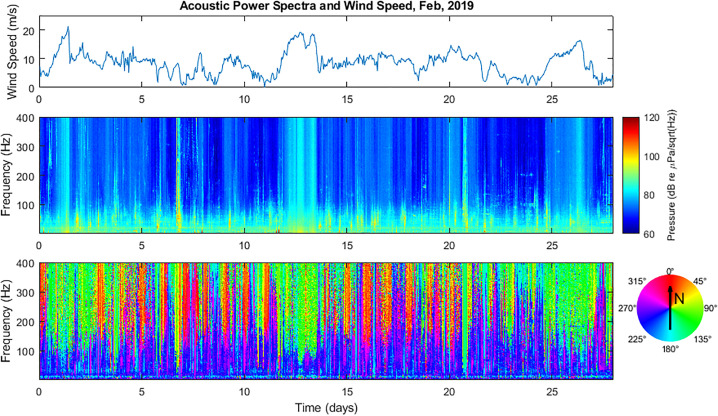
(Color online) Combined plots displaying local wind speed (upper panel), acoustic power spectra (middle panel), and directional spectra (lower panel) for the month of February 2019. High wind speeds are seen to correspond to regions of high ambient noise.

Wind speed data is collected on the NOAA buoy station 46042 about 20 km WNW of the MARS site.^16^ Figure 3 displays three subplots together for the month of February 2019. The upper subplot displays wind speed, while the middle plot displays acoustic power spectra and the lower plot displays acoustic directional spectra throughout the month. The frequency range displayed corresponds to 25–400 Hz. There is a clear correlation between the wind speed and the ambient noise levels above 100 Hz.

Although wind noise is not generally considered to be a dominant factor at frequencies below 100 Hz, it is also important to note that strong weather events cause an increase in spectral levels down to nearly 50 Hz. This is most clearly seen in the directional spectrogram data during periods of high wind, especially in excess of 15 m/s. It is worth noting that this appears consistent with breaking wave saturation, which begins to occur at wind speeds exceeding 15–20 m/s.^7^ Thus, annual weather patterns may have a noticeable impact on average acoustic spectral levels measured at the MARS/NPS-3D sensor down to nearly 50 Hz. It appears that frequencies below 50 Hz are unaffected by such climatological effects.

Analysis of the wind data for February 2019 and 2020 shows that February 2020 exhibited less significant weather events. Specifically, the average wind speeds were 8.34 and 6.33 m/s for February 2019 and 2020, respectively, and the peak wind speeds for each month were approximately 21 and 15 m/s, respectively. This could also contribute to lower acoustic spectral averages observed in February 2020 compared with 2019. Such effects make it more challenging to associate reduced acoustic energy levels only with reduced maritime activity.

### C. Spectral probability density

In order to compare long time-series spectra and signal levels, probabilistic statistics are preferred over simple averages. Following the approach outlined by Merchant *et al.*^17^ to compute spectral probability densities, the previously described power spectra were combined by averaging acoustic power, initially sampled every 0.5 s, over a 60 s time window for each 1 Hz bin between 25 and 400 Hz. This results in 1440 time samples per day over 376 frequency bins. Month-long data records were then comprised of over 40 000 time samples at 60 s spacing over 376 frequency bins with 1 Hz resolution. Histograms of 0.1 dB re 1 *μ*Pa/sqrt(Hz) bin widths were subsequently computed within each 1 Hz frequency bin to produce empirical probability densities of spectral levels. Specific results are then extracted at the 10th, 25th, 50th, 75th, and 90th percentiles of monthly acoustic spectral density levels.

**FIG. 4.**
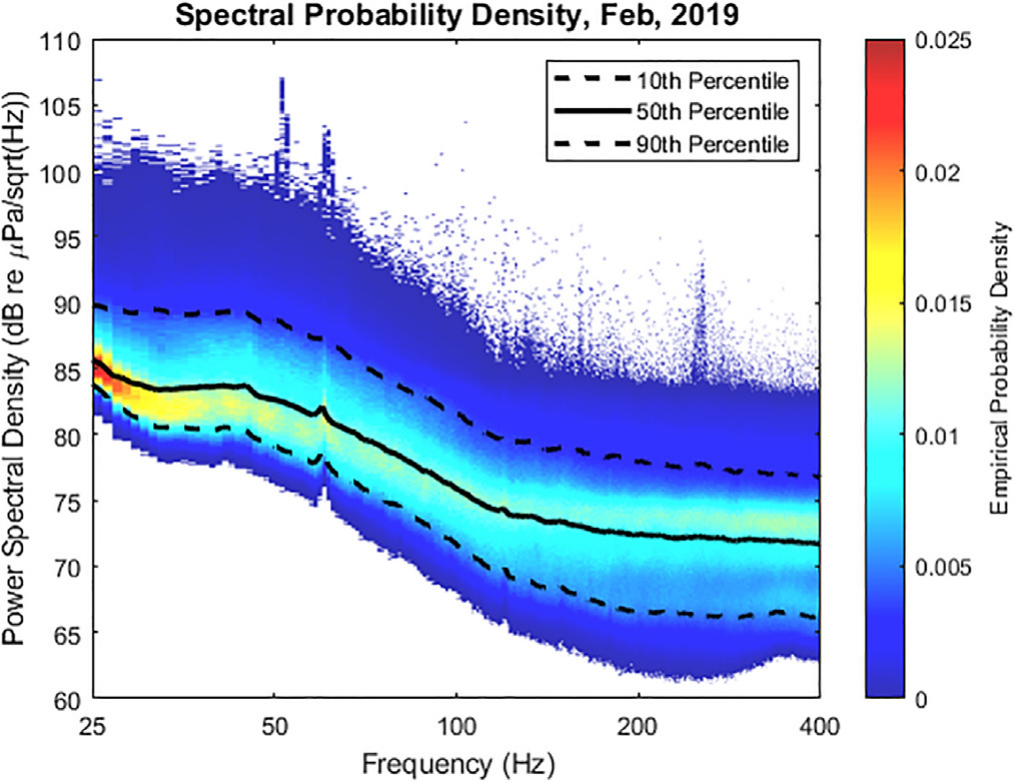
(Color online) Spectral probability density plots for the month of February 2019. The 10th, 50th, and 90th percentile curves are overlaid.

**FIG. 5.**
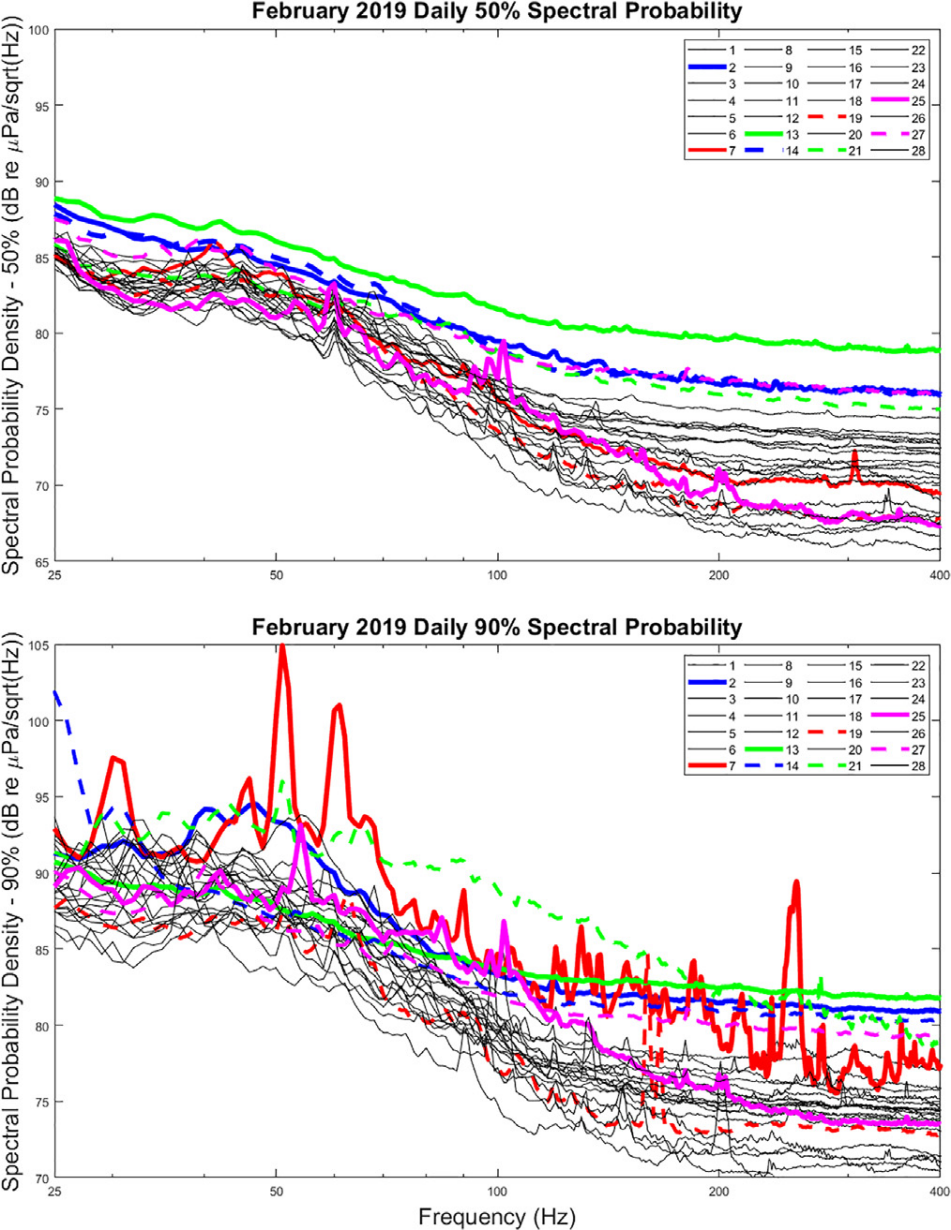
(Color online) Daily spectral probability curve comparisons at the 50th (upper) and 90th (lower) percentiles for the month of February 2019. Bold lines indicate days with high wind.

Figure 4 displays these spectral probability densities (SPDs) for the month of February 2019. Computed over an entire month, these data expose a small 60 Hz contribution as well as a 120 Hz harmonic. For a more granular view, Fig. 5 displays the 50th and 90th percentile SPD for each day in February 2019. In addition, the curves associated with certain high 50th percentiles are presented in bold colors, with corresponding bold colors added to the 90th percentile plot. A few things are worth noting in these data.

(1) The 50th percentile SPDs for February 2, 13, 14, and 27 appear to have noticeably larger levels across the entire band than other days. This is particularly true for February 13, 2019. When reviewing the results presented in Fig. 3, these days correspond to high-wind events, with wind speeds exceeding 15 m/s (approximately 30 kn) for extended periods. Such wind events are therefore seen to introduce significant low-frequency noise, even below 50 Hz.
(2) These same days do not necessarily introduce significant low-frequency noise at the 90th percentile, although they do exhibit increases above 100 Hz.
(3) On February 7 and 21, 2019, there are broadband events that lead to significant 90th percentile levels, which do not appear to be significant at the 50th percentile SPD. These were subsequently determined to be caused by research vessel operations over MARS on that day.

**FIG. 6.**
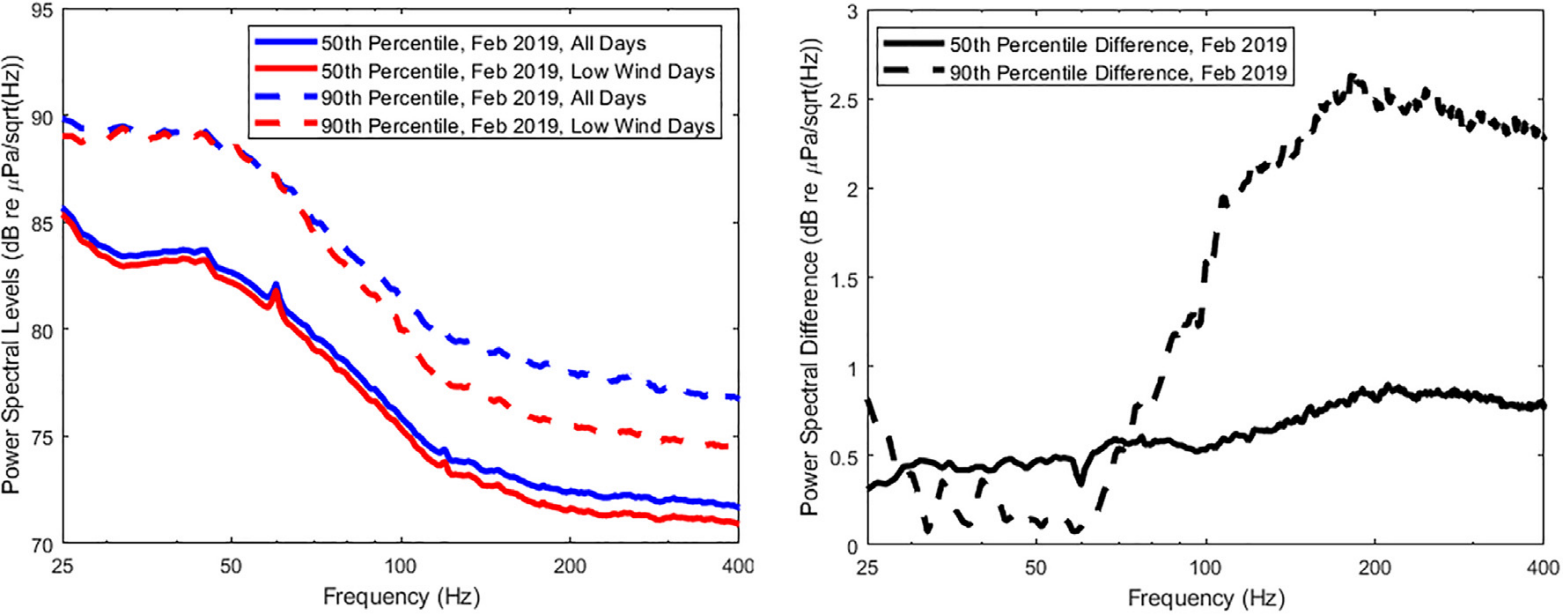
(Color online) Spectral probability density comparisons at the 50th and 90th percentiles for the month of February 2019. Left plot compares SPD curves with and without high wind days, while right plot displays differences between curves.

In order to examine the impact that these high wind events have on the statistics over the course of a month, the data were recomputed after removing those specific days from the record. Figure 6 displays the differences between the computed statistics when data from all days are used versus the exclusion of high wind event days. It is observed that, although a day-to-day comparison shows increased levels at low frequency by 3 dB or so, the month-long statistics generally show no more than 0.5 dB sensitivity below 80 Hz. Interestingly, the 90th percentile results are less affected by the high wind events below 80 Hz, presumably due to being saturated by the research vessel activity on February 7, but clearly show much more sensitivity above 100 Hz, where wind noise and breaking waves are expected to have a big impact.

The results of this detailed analysis of spectral probability density suggests that month-long averages are useful in obtaining statistical measures of signal levels below 80 Hz, even when high wind events are included in the analysis. A strong storm season may affect the results at these lower frequencies but could be expected to be limited to about 0.5 dB variation.

### D. Annual variations

**FIG. 7.**
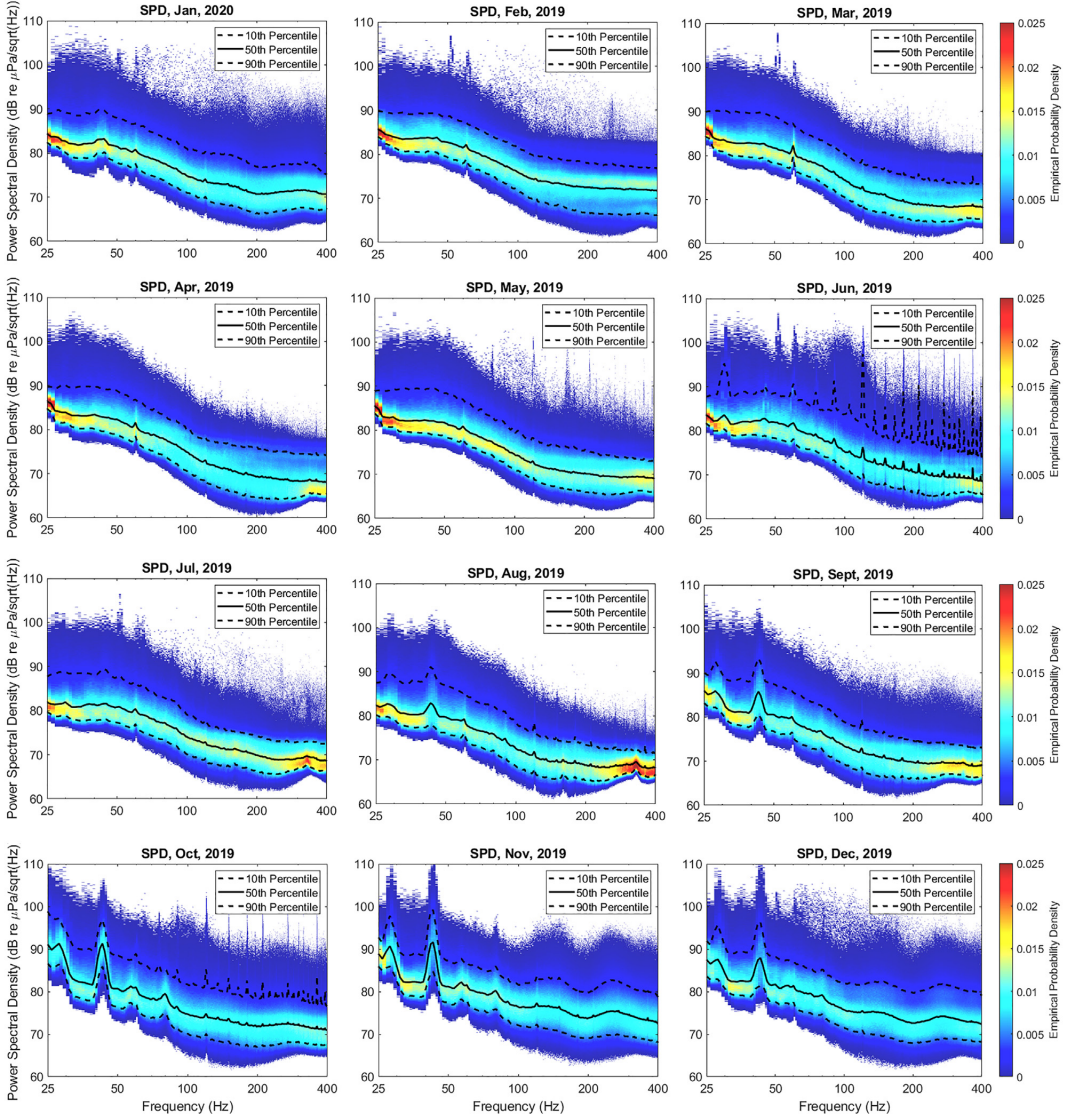
(Color online) Annual variations in SPDs, highlighting blue whale vocalizations between the months of August through December at 28 and 43 Hz.

Month-long spectral probabilities were then computed for an entire year to examine annual variations in statistics. Figure 7 displays a year's worth of data (February 2019 through January 2020) computed in the same manner as previously described. In each plot, the 10th, 50th, and 90th percentile curves are also included. While most of these plots have a consistent structure (with the exception of June 2019, which had significant research vessel activity over MARS), there is a notable exception observed beginning in late August which lasts until early January. Specifically, there is a significant increase in levels observed around 43 Hz, which is seen to peak in the October–November time frame by as much as 6 dB. This strong, persistent signal corresponds to blue whale vocalizations. These results are consistent with previous work utilizing hydrophone data collected at this site.^18^

Since the goal of this paper is to ascertain whether changes in shipping activity due to COVID can be observed in long-time acoustic data analysis, it is desirable to avoid processing data that may be impacted by other factors that are not easily distinguishable. By focusing the analysis below 80 Hz, the impact of high wind events which may change from year to year can be minimized. In order to avoid biases introduced by changes in annual blue whale migrations, the data presented above suggests that the analysis should consider frequencies above 50 Hz. Consistent with international standards for characterization of shipping noise,^19^ the remainder of the acoustic analysis presented here focused on the 1/3-octave band centered at 63 Hz (band 18, 56.2–70.8 Hz).

### E. Local AIS data

Automatic Identification System (AIS) data on marine vessel traffic in the Monterey Bay area are collected through a variety of sources, including AIS antennae mounted at sites managed by NPS. These data are combined with similar data collected by the U.S. Coast Guard and a repository is maintained at NPS. This allows for relatively quick reviews of vessel traffic after acoustic recordings are collected.

**FIG. 8.**
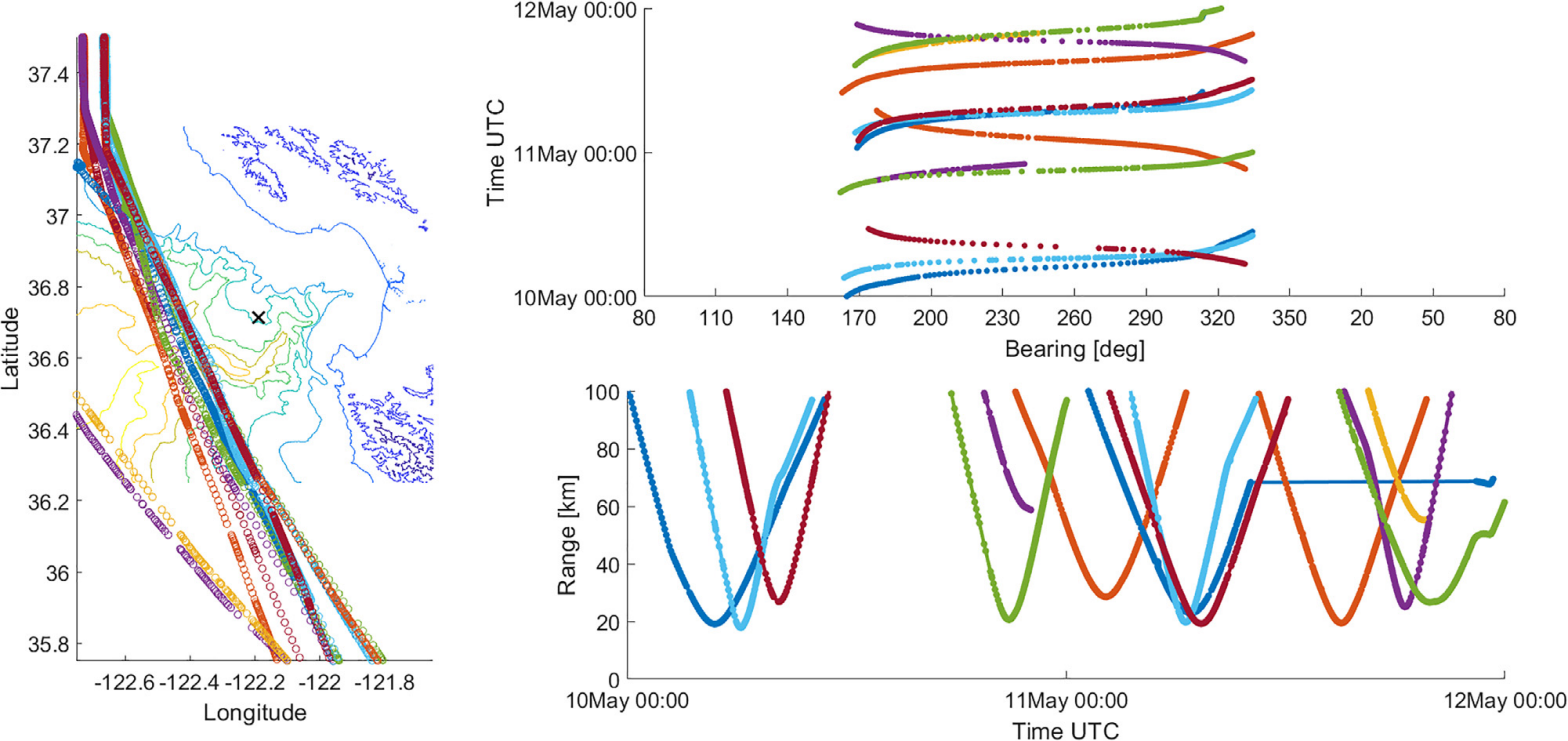
(Color online) sample AIS data for merchant vessels west of Monterey Bay from May 10 to 12, 2019. Left-most plot shows ship traffic relative to sensor location, upper right plot shows corresponding data in bearing versus time, and lower right plot shows corresponding data in range versus time.

Processing AIS data relative to the MARS/NPS-3D sensor generates relative bearings and ranges from merchant vessels to the sensor. In Fig. 8, a sample set of AIS data is presented for a 2-day period in May 2019. In this figure, only results for vessels passing to the west of the sensor are presented, which captures traffic in the shipping lanes. A complete review of AIS data also reveals many smaller vessels (e.g., fishing boats, whale-watching boats, etc.) in and around Monterey Bay. However, these tend to stay closer to the coast in shallower waters, and do not introduce significant energy on the sensor at the lower frequencies of interest. This was evident in Figs. 2 and 3, previously presented.

**FIG. 9.**
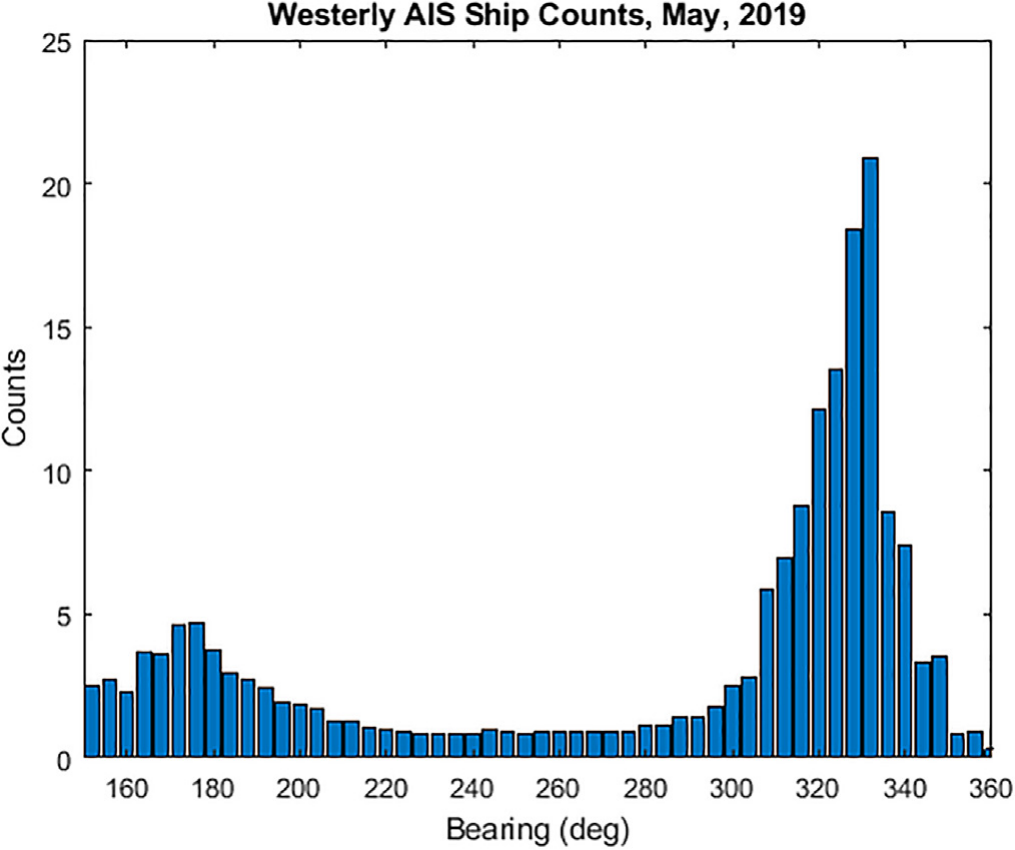
(Color online) Histogram of sample AIS data for merchant vessels west of Monterey Bay. This typical pattern shows more vessel counts to the northwest and southwest, consistent with time spent in those bearings.

A simple histogram of these westerly ship counts as a function of bearing is presented in Fig. 9. Assuming AIS data are sampled equally in time, the histogram counts represent a measure of the amount of time that vessels are within a particular bearing increment. Figure 9 exhibits a bimodal shape with peaks between about 170º and 180º to the south and between 325º and 335º to the north-northwest. There are fewer ship counts to the west near CPA simply because vessels spend less time there as they transit along the shipping lanes. The peaks to the south show a greater spread due to more variability in southerly ship transects, while the larger peaks to the north-northwest are consistent with ships grouping together as they approach the entrance to the San Francisco Bay. These same bearing highlights will be observed later in the directional acoustic data, as well.

### F. Directional processing

The MARS/NPS-3D system is a directional acoustic sensor capable of determining bearing to sources of detected signals. There are several ways to process acoustic vector sensor data in order to estimate bearing, each with pros and cons. The three approaches used here include multiplicative intensity processing, cumulative linear coherent processing, and adaptive coherent processing. In each case, only the two-dimensional horizontal plane is considered since the measure of interest is the horizontal bearing.

Bearing estimation from intensity processing is a simple method based on the ratio between horizontal components of the active acoustic intensity vector. Specifically, the complex acoustic intensity is defined by

$$\vec{J}=\frac{1}{2}p{\vec{v}}^{*}=\vec{I}+i\vec{Q},$$ (1)

where p is the complex acoustic pressure, $\vec{v}$ is the associated complex acoustic particle velocity, and $\vec{I}$ and $\vec{Q}$ represent the active and reactive portions of the complex acoustic intensity.^14^ Each of these quantities is defined in the frequency domain and can be computed for every time/frequency bin in the complex spectrogram. Estimates of horizontal bearing are then computed simply according to

$$\theta ={\text{tan}}^{-1}\left(I_{y}/I_{x}\right),$$ (2)

where the coordinate system is defined such that the positive x-direction is aligned with north and the positive y-direction is aligned with east. This type of calculation was employed in the generation of the directional spectrograms in Figs. 2 and 3.

Linear coherent processing is a form of conventional beamforming (CBF) in which the different acoustic channels (pressure and particle velocity) are scaled by appropriate weighting and phase factors corresponding to different steering angles, then summed coherently to form peaks in the direction that best matches the incoming signal. For a single vector sensor, the linear beamformer output may be defined as^20^

$$B\left(\theta_{s}\right)={\left|\left[p+w_{x}\left(\theta_{s}\right)v_{x}+w_{y}\left(\theta_{s}\right)v_{y}\right]\right|}^{2},$$ (3)

where

$$w_{x}\left(\theta_{s}\right)=A\rho c\;\text{cos}\;\theta_{s}\;\text{and}\;w_{y}\left(\theta_{s}\right)=A\rho c\;\text{sin}\;\theta_{s}.$$ (4)

The plane wave acoustic impedance factor ρc is included in the weighting to scale all acoustic terms in units of pressure, while the factor A scales the directional components relative to the omnidirectional pressure response. When A=1, this CBF approach produces standard cardioid patterns in the presence of plane waves.

A more general definition of the CBF response in Eq. (3) may be represented by^21,22^

$$B_{\mathrm{CBF}}\left(\theta_{s}\right)={\vec{w}}^{†}\left(\theta_{s}\right)K\vec{w}\left(\theta_{s}\right).$$ (5)

The vector $\vec{w}$, often referred to as the plane wave replica vector, is defined (with A=1) by

$$\vec{w}=\left[\begin{array}{c} 1\\ \rho c\;\text{cos}\;\theta_{s}\\ \rho c\;\text{sin}\;\theta_{s} \end{array}\right],$$ (6)

and the matrix K represents the cross-spectral data matrix (CSDM), defined by

$$K=\left[\begin{array}{ccc} pp^{*} & pv_{x}^{*} & pv_{y}^{*}\\ v_{x}p^{*} & v_{x}v_{x}^{*} & v_{x}v_{y}^{*}\\ v_{y}p^{*} & v_{y}v_{x}^{*} & v_{y}v_{y}^{*} \end{array}\right],$$ (7)

where the superscripts “*” and “†” refer to complex conjugate and complex conjugate transpose, respectively. Utilizing this more general formulation, the response of the minimum variance distortionless response (MVDR) adaptive processor can be shown to have the form^21,22^

$$B_{\mathrm{MVDR}}={\left[{\vec{w}}^{†}\left(\theta_{s}\right)K^{-1}\vec{w}\left(\theta_{s}\right)\right]}^{-1}.$$ (8)

For the bearing estimation results that follow, the previously described 60-s coherently averaged complex spectrogram data were utilized to form the various elements of the CSDM defined in Eq. (7). These data were then further coherently averaged over the 1/3 octave band centered at 63 Hz. From that, the intensity processing results can be obtained from the real parts of $pv_{x}^{*}$ and $pv_{y}^{*}$. The CBF and MVDR results follow directly from Eqs. (5) and (8), respectively.

**FIG. 10.**
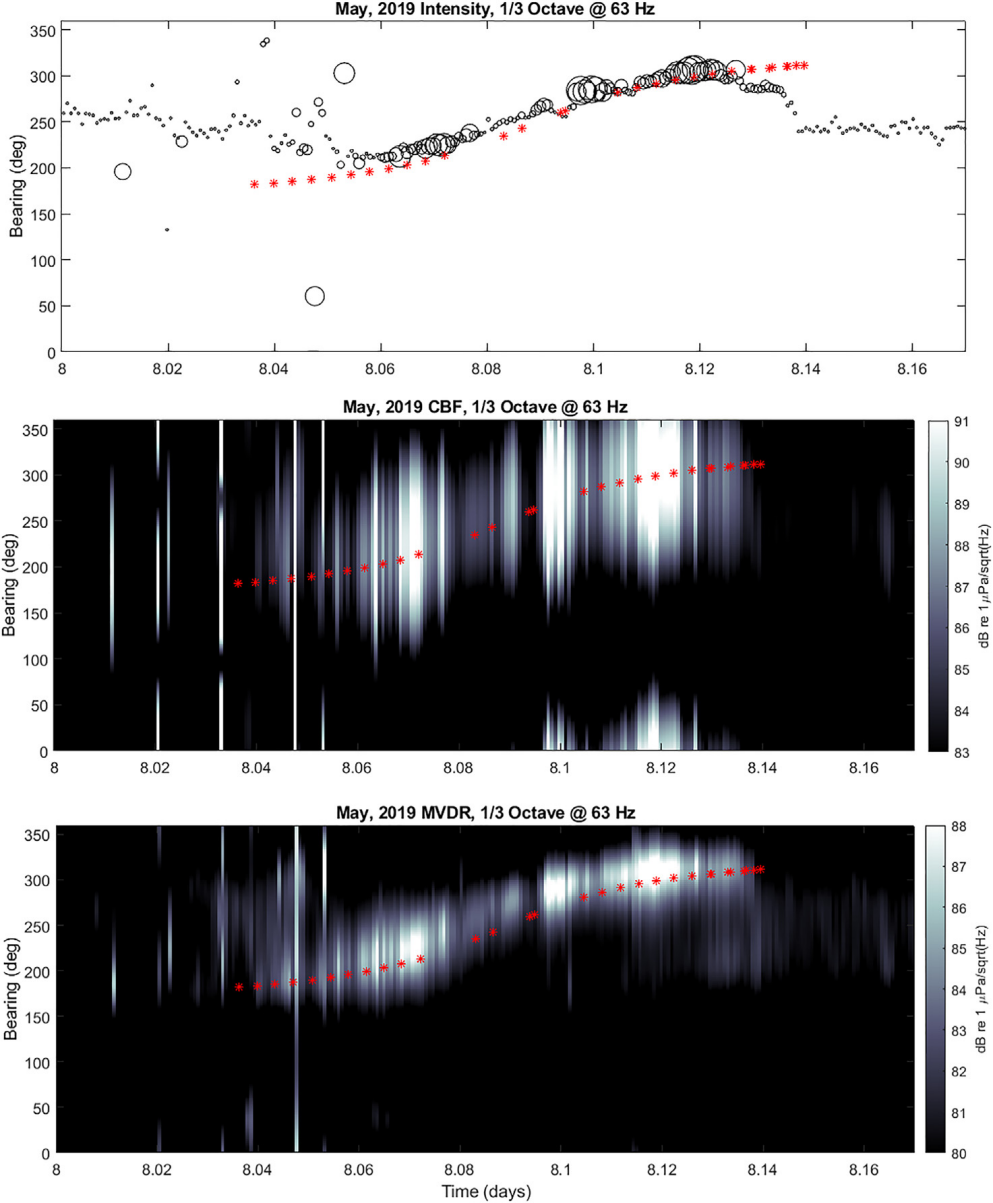
(Color online) sample bearing estimate results for 1/3 octave centered at 63 Hz from May 9, 2019 during passage of the single merchant vessel Aegean Highway. Upper panel displays intensity processing results {the size of the circles have been scaled to represent the relative level of the acoustic intensity with the smallest circles corresponding to anything ≤80 dB [re 1 *μ*Pa/sqrt(Hz)] and the largest corresponding to anything ≥90 dB [re 1 *μ*Pa/sqrt(Hz)]}; middle panel displays CBF results; lower panel displays MVDR results. Red asterisks indicate true bearings of merchant and good tracking by all three methods.

A comparison of the results from these three different directional processing methods is provided in Fig. 10 during the passage of a single merchant vessel, the Aegean Highway, on May 9, 2019. The intensity processing simply produces a single value for the bearing estimate which are plotted as circles in the upper panel. The size of the circles have been scaled to represent the relative level of the acoustic intensity with the smallest circles corresponding to anything ≤80 dB [re 1 *μ*Pa/sqrt(Hz)] and the largest corresponding to anything ≥90 dB [re 1 *μ*Pa/sqrt(Hz)]. The scales on the CBF and MVDR results are also in units of dB [re 1 *μ*Pa/sqrt(Hz)]. In addition, on each panel are plotted red “+” symbols corresponding to the bearing computed from the AIS data.

Each of the results show favorable agreement with the AIS bearing data, indicating that bearing can be accurately estimated from a single sensor in the presence of positive signal-to-noise ratio (SNR). Typically, it is noted that an SNR > 1 dB is sufficient to produce an accurate bearing estimate within about +/− 5 deg. As expected, the MVDR response produces a narrower peak than CBF, although the peaks of both appear along the same bearings.

**FIG. 11.**
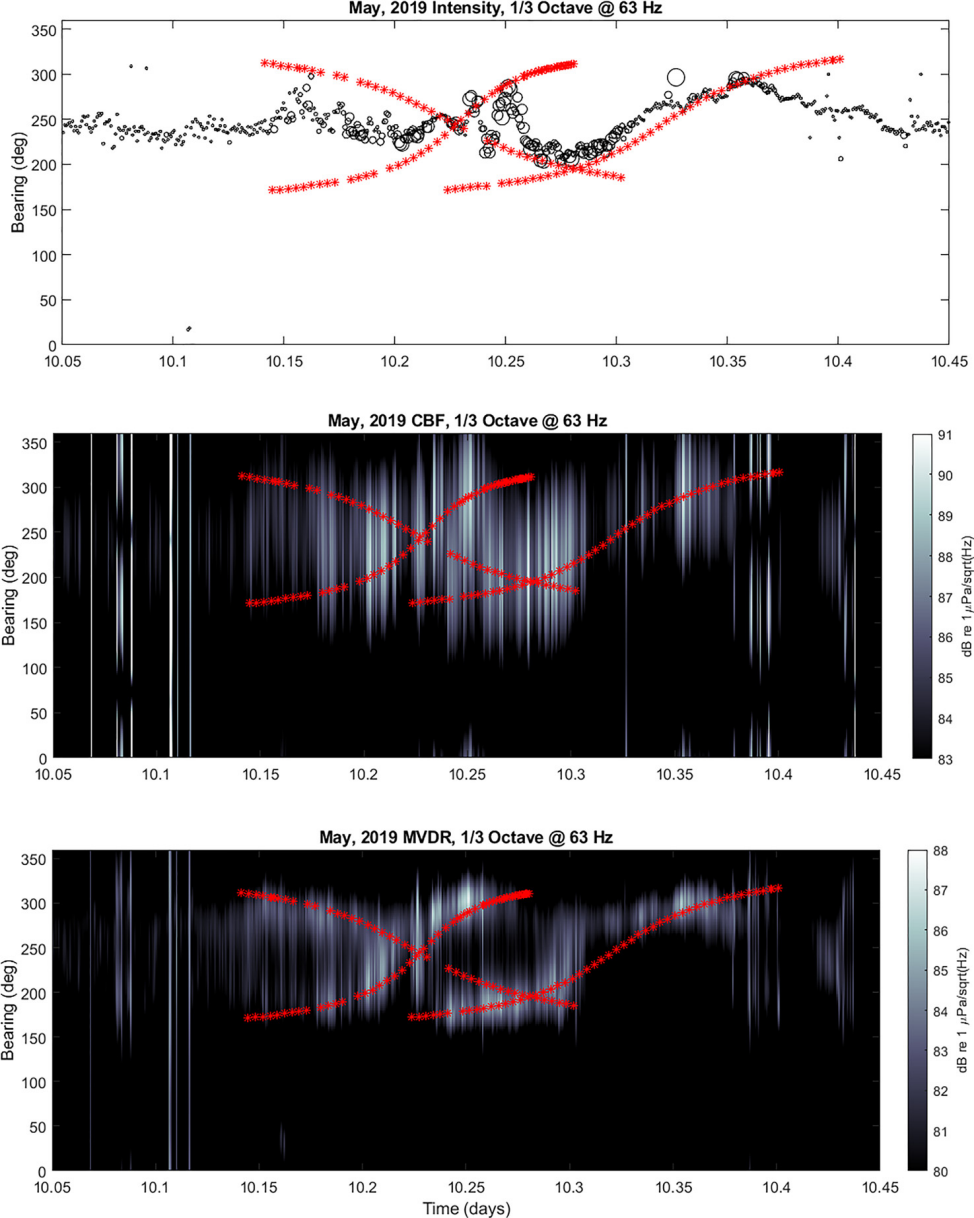
(Color online) sample bearing estimate results for 1/3 octave centered at 63 Hz from May 11, 2019 during passage of three merchant vessels: CAP Pasley, Hyundai Hong Kong, and Sara Leader. Upper panel displays intensity processing results {the size of the circles have been scaled to represent the relative level of the acoustic intensity with the smallest circles corresponding to anything ≤80 dB [re 1 *μ*Pa/sqrt(Hz)] and the largest corresponding to anything ≥90 dB [re 1 *μ*Pa/sqrt(Hz)]}; middle panel displays CBF results; lower panel displays MVDR results. Red asterisks indicate true bearings of merchant and interference observed in intensity and CBF processing, while MVDR is able to distinguish interferers.

A limitation of intensity processing or CBF from a single sensor becomes evident in the presence of in-band interfering sources, as evidenced in Fig. 11 during the passage of three merchant vessels, the CAP Pasley, Hyundai Hong Kong, and Sara Leader, on May 11, 2019. In this case, the intensity processing and CBF methods both produce a single peak response that is the average of the true bearings. This is due to the coherent interference between the signals on a single vector sensor that share a common frequency response.

Fortunately, MVDR processing on a single vector sensor is capable of distinguishing two interfering signals. A two-dimensional vector sensor measures three acoustic quantities: acoustic pressure and horizontal components of particle velocity. For a vector sensor at position ${\vec{r}}_{0}$ and a plane wave incident along azimuthal bearing θ, the output of these measurements in the absence of noise may be generally defined as

$$\begin{array}{l} p\left({\vec{r}}_{0},t\right)=P\left(\theta \right)e^{i\left(\vec{k}\cdot {\vec{r}}_{0}-\omega t\right)},\\ v_{x}\left({\vec{r}}_{0},t\right)=V_{x}\left(\theta \right)e^{i\left(\vec{k}\cdot {\vec{r}}_{0}-\omega t\right)},\\ v_{y}\left({\vec{r}}_{0},t\right)=V_{y}\left(\theta \right)e^{i\left(\vec{k}\cdot {\vec{r}}_{0}-\omega t\right)}. \end{array}$$ (9)

Allowing the location of the sensor to be at the origin, noting that $P(\theta )=P_{0}$ (omnidirectional), $V_{x}(\theta )=V_{0}\;\text{cos}\;(\theta )$, $V_{y}(\theta )=V_{0}\;\text{sin}\;(\theta )$, and using the plane wave relationship $P_{0}=\rho cV_{0}$ to define an equivalent velocity term $v_{p}\left(t\right)=p\left(t\right)/\rho c$, the output of the sensor may be written

$$\begin{array}{l} v_{p}(0,t)=V_{0}e^{-i\omega t},\\ v_{x}(0,t)=V_{0}\;\text{cos}\;(\theta )e^{-i\omega t},\\ v_{y}(0,t)=V_{0}\;\text{sin}\;(\theta )e^{-i\omega t}. \end{array}$$ (10)

These three equations can be augmented with an additional equation, specifically $\;{\text{cos}}^{2}(\theta )+{\text{sin}}^{2}(\theta )=1$, yielding four equations with three unknowns, $\left\{V_{0},\theta ,\omega \right\}$, which is theoretically solvable—though the equations are non-linear.

If we now consider two incident plane waves of common frequency, then this becomes

$$\begin{array}{l} v_{p}\left(t\right)=S_{1}e^{-i\omega t}+S_{2}e^{-i\omega t},\\ v_{x}\left(t\right)=S_{1}C_{x1}e^{-i\omega t}+S_{2}C_{x2}e^{-i\omega t},\\ v_{y}\left(t\right)=S_{1}C_{y1}e^{-i\omega t}+S_{2}C_{y2}e^{-i\omega t}, \end{array}$$ (11)

where $C_{x}\left(\theta \right)=\text{cos}\;\left(\theta \right)$, $C_{y}\left(\theta \right)=\text{sin}\;\left(\theta \right)$, and $\left\{S_{1},S_{2}\right\}$ may be complex-valued. The number of known quantities (which are measured) or equations are

{Re[vp],Im[vp],Re[vx],Im[vx],Re[vy],Im[vy]}

as well as $C_{x1}^{2}+C_{y1}^{2}=1$ and $C_{x2}^{2}+C_{y2}^{2}=1$, for a total of eight knowns. The number of unknowns are

{Re[S1e−iω1t],Im[S1e−iω1t],Re[S2e−iω2t],Im[S2e−iω2t],Cx1,Cx2,Cy1,Cy2}

or eight unknowns and eight equations. In general, for *M* sources and *N* two-dimensional vector sensors, the number of knowns is

(N sensors) × (3 equations × 2(Real and Imaginary))+M trigonometric identities=6N+M.

The number of unknowns is

(M sources)×[2(Real and Imaginary components)+2 (direction cosines)] = 4M.

Hence, the number of sensors required to estimate bearings to *M* sources is

6N+M≥4M⇒6N≥3M⇒2N≥M. (12)

This argument can be made for three-dimensional sensors estimating directions towards sound sources anywhere in space, and the result is the same. Theoretically, in the absence of noise and with ideal sensors, *N* number of vector sensors can distinguish the direction of arrival of 2 *N* incoming signals of coincident frequency. A similar issue was addressed by Hickling and Morgan^23^ for intensity processing of two non-coherent sources.

The lower panel of Fig. 11 confirms this with the resolution of two distinct peaks during times of interfering signals. It is worth noting that these results confirm that passing merchant vessels spend more time in bearings concentrated to the south and to the northwest. Additionally, by comparing the times when the directional processing is able to accurately estimate bearings, the maximum range at which these vessels can be tracked tends to be approximately 50–60 km.

Finally, it can also be observed in many cases, especially in the presence of a single vessel transit, that there is a fading of the acoustic intensity near the CPA. This is counter-intuitive since CPA corresponds to the shortest distance between the vessel and the sensor. It is speculated that this phenomenon is due to the specific propagation conditions in the area, which are highly influenced by the local bathymetry.

Due to the ability of the MVDR processor to determine directional energy distributions, including the separation of up to two interfering contacts, those results are then further analyzed over month-long time periods to develop noise level statistics.

To generate long-term statistics for year-to-year comparison, MVDR beams were calculated for all 1-Hz frequency bins in each 60-s data snapshot with look directions every 2°. All spectral averages within the limits of the 63 Hz 1/3 octave band were combined to produce an average spectral level in each direction for each snapshot. The average spectral level for each snapshot in each month was tallied to generate empirical CDFs for the beam amplitude in each look direction. For computing the CDF, amplitudes were split into 0.1 dB bins from 40 to 140 dB re 1 *μ*Pa/sqrt(Hz), with bin counts scaled for probability such that the sum of each CDF across all bins equaled 1. A similar process was performed to calculate CDFs for the omnidirectional pressure channel for each month.

**FIG. 12.**
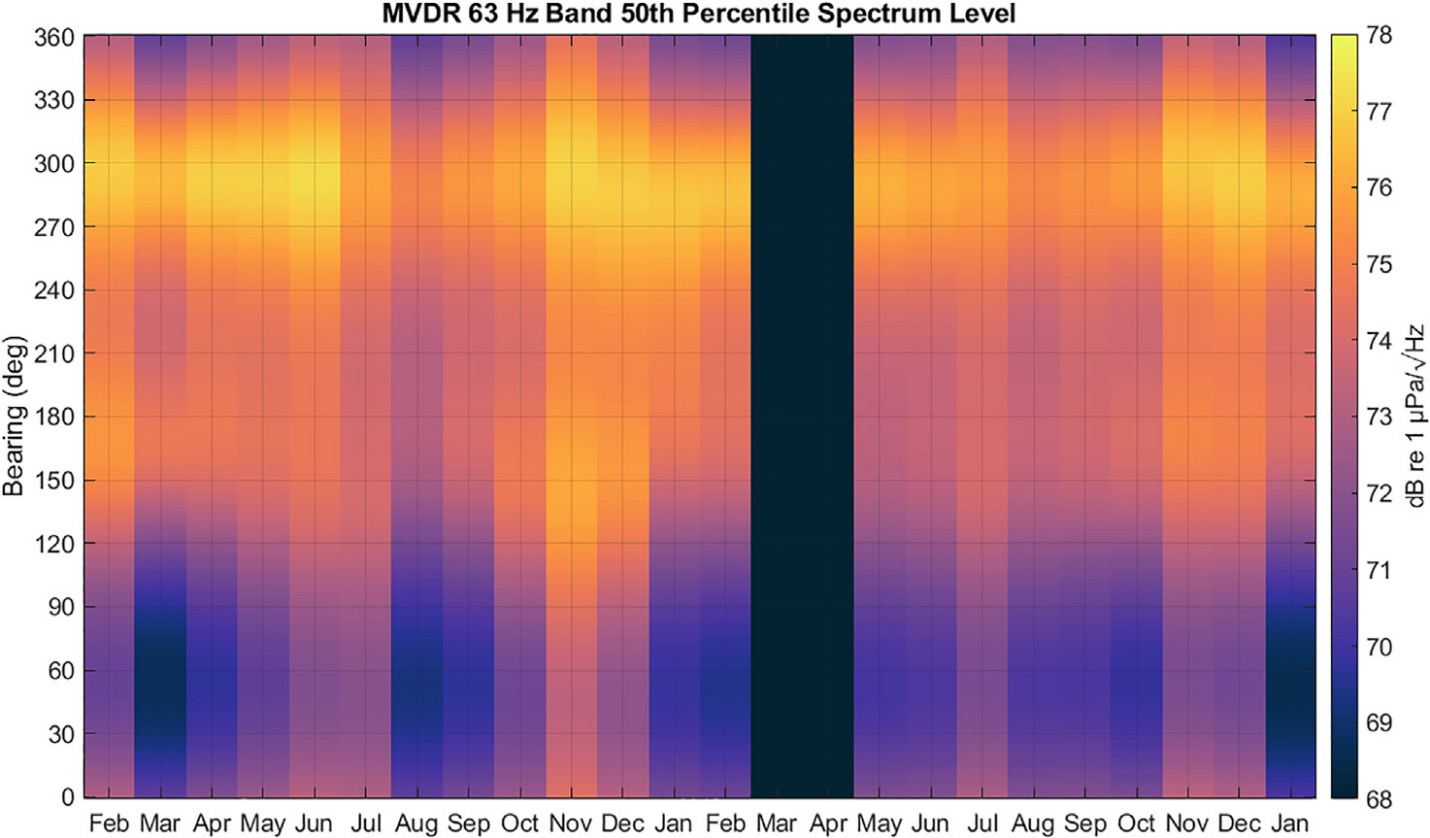
(Color online) 63 Hz 1/3 octave band MVDR 50th percentile spectrum level computed every month from February 2019 to January 2021. Peaks are observed near 290º and 170°, consistent with time spent in those bearings by distant shipping.

Figure 12 shows an example of 50th percentile data extracted from each CDF in each look direction for all the months. The data showed a robust bimodal response, with a well-defined primary peak centered around 290° and a lesser-defined but consistent secondary peak centered at 170°. These two directions were selected for further year-to-year comparisons, along with the omnidirectional channel. The increase in noise level across all look directions in November and December in both years relative to the months before and after them is attributed to vocalizations from migrating blue whales.

## III. FINAL RESULTS AND OBSERVATIONS

Two categories of AIS records were removed prior to analysis. Records having positions over land or within San Francisco Bay were removed using the *inpolygon* function of the *pracma* package for r (version 3.6.3) with a land polygon mask defined by full-resolution GSHHS coastline data. Redundant records were removed by requiring that a vessel, identified by its Maritime Mobile Service Identity (MMSI) number, be represented only once in each 5-min data summary. Vessel length was computed by adding the AIS data fields that quantify the distances between the AIS transmitter and the vessel's bow and stern.

The two-year AIS data time-series, comprising around 3.3 × 10^6^ records within the direction range outside Monterey Bay (135° to 360°), was analyzed for comparison with the directional acoustic data. Vessel presence was summed within 10° directional bins, after each record was weighted according to three factors: acoustic transmission loss (TL), vessel speed, and vessel length. TL between source locations and MARS were computed using the RAM parabolic equation model.^24^ Source depth was specified as 6 m, and source frequency was specified as 63 Hz to be consistent with acoustic analysis. The model domain extended 165 km from the receiver. Specification of regional ocean temperature and salinity was based on the January climatology from the U.S. Navy Generalized Digital Environmental model (GDEM). Bathymetry was specified at 250 m resolution. TL weighting for each AIS record was assigned by cross-referencing TL results and AIS data in a common discrete global grid using the dggridR (version 2.0.8) toolbox in r (version 4.1.0). Consistent with weighting by linear-scale transmission loss, the weighting factor was 10^[–(TL–TL*min)/*10]^, scaled such that the minimum TL within the model domain (near the hydrophone) was assigned a weighting value of 1 and all other TL values were assigned weighting values below 1.

Weighting by vessel speed applied a statistical model developed using nearly 600 examples of recorded container vessel transits, which showed that vessel speed had the greatest predictive power for noise across the full frequency range examined, 20–1000 Hz.^25^ According to this model, vessel noise source level (SL) is a quasi-exponential function of vessel speed. In the present study, each record of vessel presence was weighted according to vessel speed using the published model function for the octave band centered at 63 Hz. Records having unreasonably high vessel speeds, >13 m/s (25.2 kn, < 0.1% of records) were excluded. The final weighting factor was vessel length, applied as a linear scale factor. Using all three scaling factors, each vessel position record was converted to the product of all three scale factors. These transformed values were summed within each directional bin and normalized by the maximum for representation relative to the directional acoustic statistical summary.

**FIG. 13.**
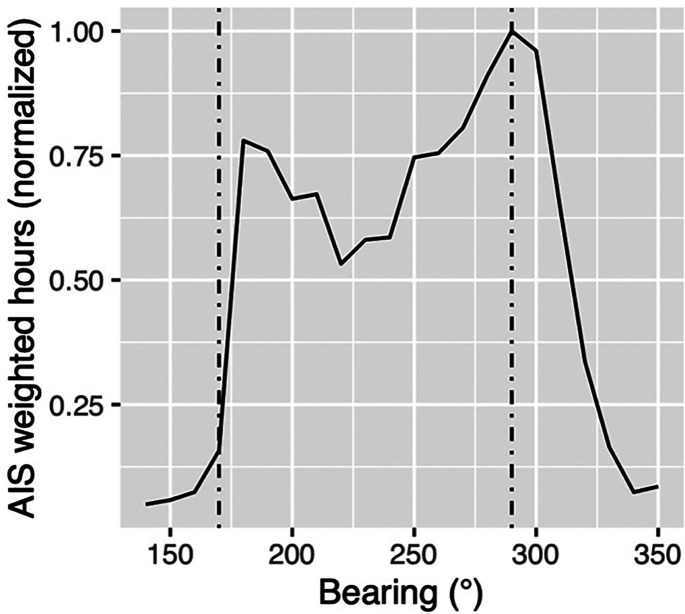
Predicted 63 Hz received signal level weighted distribution (normalized) of merchant vessels as a function of bearing angle (solid line). Two dashed lines indicate dominant observed signal level bearings from MVDR processing.

The results of this weighted distribution as a function of bearing are presented in Fig. 13, along with the observed peak signal bearings from the previous MVDR processing at 63 Hz. It is worth noting that this predicted distribution agrees very well with the peak observed in the NW direction. The peak to the SW is also close, but the observation appears slightly more southerly than the prediction. This could be due to azimuthal refraction off the Pt Sur ridge and nearby continental slope, as was previously speculated in Ref. 26.

**FIG. 14.**
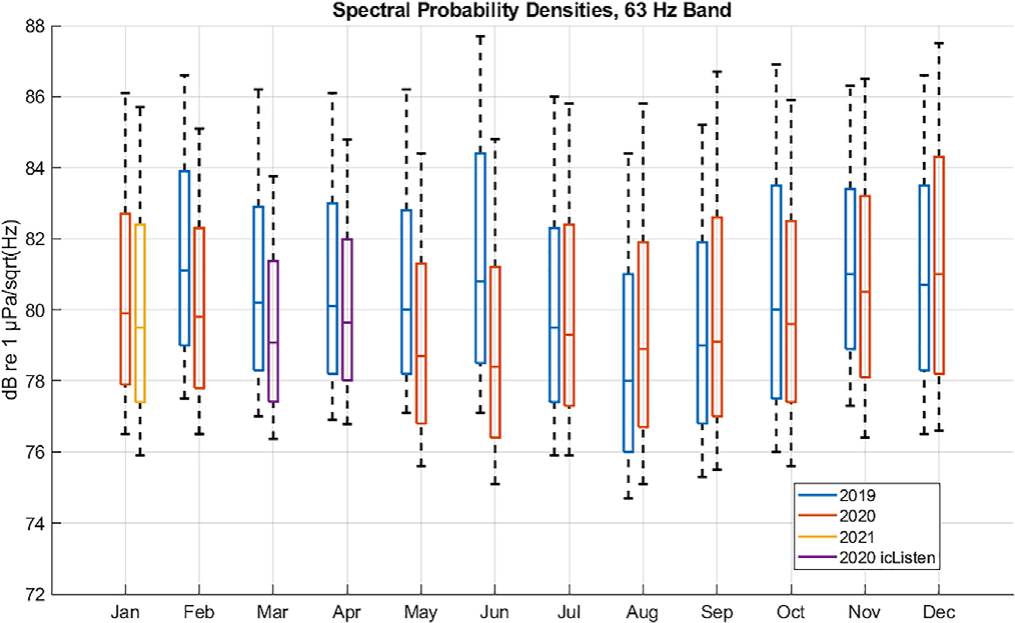
(Color online) Monthly spectral probability percentiles of omni-directional data between February 2019 and January 2021 showing year to year changes. Lower and upper whiskers represent 10th and 90th percentiles, while boxes represent 25th, 50th, and 75th percentiles. Lower levels from February through June of 2020 relative to 2019 are consistent with reduced shipping caused by COVID-19.

Utilizing the tools described above to compute month-long statistics of merchant vessel noise in the 1/3 octave band centered at 63 Hz, data over the two-year period beginning in February 2019 through January 2021 was processed. This allows for a comparison of levels between 2019 and 2020, when the COVID-19 pandemic forced changes in world-wide commerce. Figure 14 shows the year-to-year comparison of monthly spectral probability density statistics of the omnidirectional hydrophone data at the 10th, 25th, 50th, 75th, and 90th percentiles. Due to the missing NPS-3D data during the March–April 2020 time frame, hydrophone data from the nearby icListen sensor was provided for comparison. (In order to ensure proper scaling, these data were adjusted such that the levels for the other months of the year matched.)

The results in Fig. 14 display several interesting features:

(1) A noticeable drop in noise levels is first observed in February 2020, of about 1–1.5 dB.
(2) These lower values appear through May 2020, followed by the largest annual drop in noise levels during the month of June 2020, which shows a decrease of 2–2.5 dB.
(3) Beginning in July 2020, levels seem to return to pre-COVID ranges, although there is a hint of a potential increase in August 2020. This may suggest an increase in merchant vessel traffic as commerce attempts to overcome prior reductions.

**FIG. 15.**
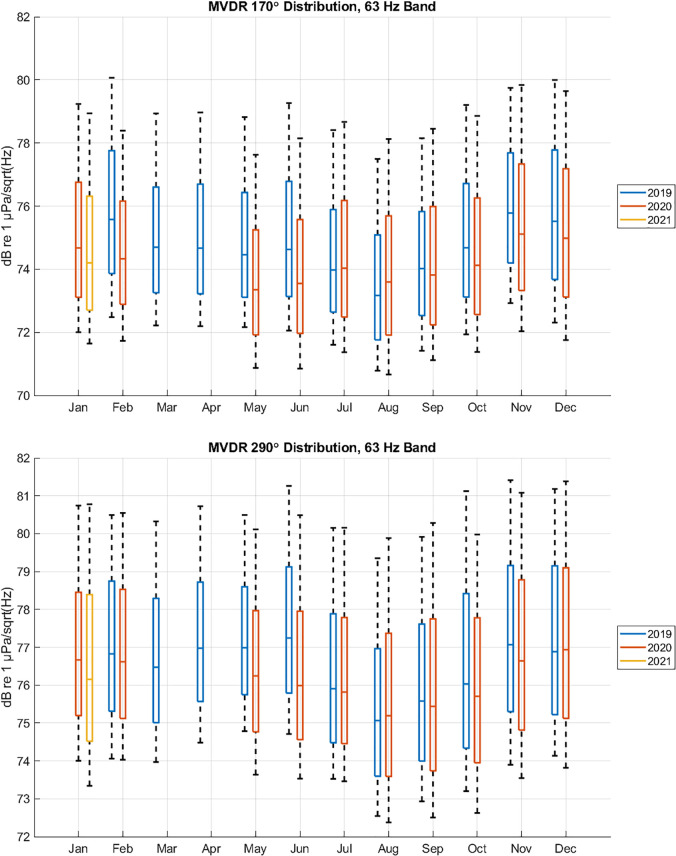
(Color online) Monthly spectral probability percentiles of MVDR directional data between February 2019 and January 2021 showing year to year changes. Upper panel presents data at bearings of 170 +/− 20º, lower panel presents data at bearings of 290 +/− 20º. Lower and upper whiskers in each panel represent 10th and 90th percentiles, while boxes represent 25th, 50th, and 75th percentiles. While both bearings show decreased levels from COVID-19 impact, the effect is more pronounced to the southwest which corresponds directly to transiting merchants.

Figure 15 displays similar statistical data extracted from the MVDR analysis by selecting 40º wide fans centered at 170º (southerly) and 290º (north-northwesterly). The general trends of these plots are consistent with those observed in Fig. 14. There are some noteworthy exceptions, however.

(1) The reduction in levels in February 2020 does not appear to the NNW, but rather is mostly seen to the S. This implies the primary reduction in noise was due to a decrease in the number of vessels transiting, which would only be seen in the southerly direction, but that the number of vessels near the entrance to the San Francisco Bay were not as affected initially.
(2) Reductions in levels between the two directions were more comparable in later months, although the differences still seem slightly more pronounced to the S.
(3) The potential increase in levels during the month of August 2020 also appears slightly more pronounced in the southerly direction, indicating a larger increase in transiting vessels in the shipping lanes.

**FIG. 16.**
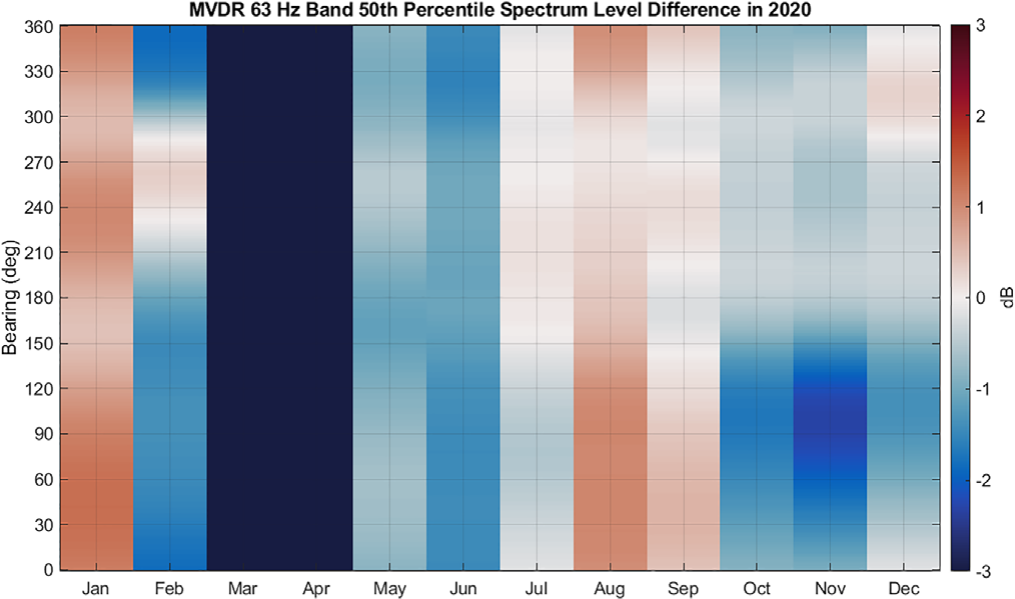
(Color online) 63 Hz 1/3 octave band MVDR 50th percentile spectrum level year-to-year differences. Red indicates an increase in noise level from 2019 to 2020, and blue indicates a decrease in noise level. January data compares 2020 to 2021, since data were not available for January 2019. Vector sensor processing reveals the directional dependence of the ambient noise changes, which is lost when measuring noise with a single hydrophone.

The trends observed in the two sectors around 290° and 170° persisted across all look directions, as shown in Fig. 16. February, May, and June showed reduced 50th percentile levels from 2019 to 2020 across all look directions, whereas sound levels returned to 2019 levels in July and even exceeded 2019 levels in August. The decreased levels in November 2019 relative to November 2020 are not in the direction of the shipping lane but are instead shoreward, in the direction of the canyon. Since this period coincides with the arrival of migrating blue whales and an overall increase in noise level compared to the preceding months, we do not attribute these changes to changes in shipping noise. The ability to compare noise changes in different directions and connect them to different sources demonstrates the value of using a vector sensor for long-term noise monitoring compared to a single, omnidirectional hydrophone.

## IV. CONCLUSIONS

Nearly two full years' worth of acoustic data were recorded on a directional acoustic vector sensor outside of Monterey Bay between February 2019 and January 2021. This paper presented the analysis of that data based primarily on the statistical measures of spectral probability densities of acoustic power spectra. The directional nature of the sensor also provided unique perspectives on the distribution of the acoustic energy recorded over that time period. Of specific interest was the impact of changes in merchant shipping traffic due to the COVID-19 pandemic on the ambient soundscape.

The long period of data recording provided some unique perspectives on soundscape variations, particularly at lower frequencies where merchant vessel noise is most prevalent. One interesting observation was the extent to which high wind events (corresponding to the breaking wave saturation limit of roughly 15 m/s) generated observable noise at frequencies down to 50 Hz or lower. This had a noticeable impact on the spectral probabilities during those events. In order to mitigate the impact of such events on year-to-year comparisons, it was necessary to compute statistics over an entire month of data. Even then, to reduce the impact to less than 1/2 dB required analysis to be limited below 80 Hz.

An examination of annual variations of month-long statistics also revealed the significant impact of marine mammal vocalizations. Specifically for the Monterey Bay area, blue whale migrations from the September through December seasons introduced considerable energy around 43 Hz. Thus, in order to focus the analysis on merchant vessel variations only, the recommendations of the International Quiet Ocean Experiment Workshop (2019) were followed using the 1/3 octave band centered at 63 Hz.^19^

By restricting the analysis to this single 1/3 octave band, comparison of the relative performance of various directional processing algorithms of the vector sensor data were achieved. This included multiplicative vector intensity processing, coherent linear (conventional) beamforming, and coherent adaptive (MVDR) beamforming. Each method was capable of accurately estimating the bearing towards a single merchant vessel source with sufficient signal strength. As expected, the vector intensity processing and linear beamforming methods suffered from directional ambiguity in the presence of in-band interferers. However, as was demonstrated, the adaptive beamforming approach was still able to discriminate bearings between two interfering targets. A simple algebraic argument was made to justify this result and was generalized to define the limit of discriminating multiple in-band interferers from multiple sensors. Future data collection efforts are still needed to validate this in practice.

The directional analysis provided the opportunity to distinguish two primary look directions associated with (1) a transit-only lane to the southwest and (2) a transit lane plus harbor approach location to the northwest. This capability permitted a distinction of changes in noise levels due to changes in transiting vessels rather than other factors impacting noise near harbor approaches (e.g., extended loitering periods due to port delays). Overall changes in noise levels observed on the omnidirectional channel were found to be more consistent with the directional levels to the southwest, indicating that transiting vessels dominate the ambient noise rather than the total number of merchant vessels in the region. Follow-up work will utilize this capability on data from the first half of 2021, when preliminary review of AIS data showed significant vessel traffic loitering to the northwest near the San Francisco Bay ingress.

Although the data analyzed in this work focused on low frequencies between February 2019 and January 2020, it is worth noting that these data sets encompass a broader range of frequencies and continue to stream data for additional analysis. Future work will examine features of the noise during high wind events, specifically the extent to which the directional data correlates to wind and/or wave directions as well as the impact of bathymetric features on noise directionality. The data also provides a plethora of information on marine mammal vocalizations and will aid in tracking algorithms that monitor migration patterns. Furthermore, it is anticipated that the current single vector sensor system will be replaced by a pair of vector sensors in early 2022, providing improved signal gain and directionality in future studies.
